# STAT3-dependent transactivation of miRNA genes following *Toxoplasma gondii* infection in macrophage

**DOI:** 10.1186/1756-3305-6-356

**Published:** 2013-12-16

**Authors:** Yihong Cai, He Chen, Lei Jin, Yibo You, Jilong Shen

**Affiliations:** 1 Anhui Provincial Laboratories of Pathogen Biology and Zoonoses, Department of Microbiology and Parasitology, Anhui Medical University, Hefei, China; 2 Department of Health Inspection and Quarantine, School of Public Health, Anhui Medical University, Hefei, China; 3 Department of Immunology, Anhui Medical University, Hefei, Anhui, China; 4 Kolling Institute, University of Sydney, Darlington, Australia; 5 Department of Microbiology and Immunology, School of Life Sciences, University of Science and Technology of China, Hefei, China

**Keywords:** *Toxoplasma*, MicroRNA, Apoptosis, STAT3, Macrophage

## Abstract

**Background:**

The apicomplexan parasite *Toxoplasma gondii* can infect and replicate in virtually any nucleated cell in many species of warm-blooded animals; *T. gondii* has elaborate mechanisms to counteract host-cell apoptosis in order to maintain survival and breed in the host cells.

**Methods:**

Using microarray profiling and a combination of conventional molecular approaches, we investigated the levels of microRNAs (miRNAs ) in human macrophage during *T. gondii* infection. We used molecular tools to examine *Toxoplasma*-upregualted miRNAs to revealed potential signal transducers and activators of transcription 3(STAT3) binding sites in the promoter elements of a subset of miRNA genes. We analysed the apoptosis of human macrophage with the functional inhibition of the STAT3-binding miRNAs by flow cytometry.

**Results:**

Our results demonstrated differential alterations in the mature miRNA expression profile in human macrophage following *T. gondii* infection. Database analysis of *Toxoplasma*-upregulated miRNAs revealed potential STAT3 binding sites in the promoter elements of a subset of miRNA genes. We demonstrated that miR-30c-1, miR-125b-2, miR-23b-27b-24-1 and miR-17 ~ 92 cluster genes were transactivated through promoter binding of the STAT3 following *T. gondii* infection. Importantly, functional inhibition of selected STAT3-binding miRNAs in human macropahges increased apoptosis of host cells.

**Conclusions:**

A panel of miRNAs is regulated through promoter binding of the STAT3 in human macrophage and these miRNAs are involved in anti-apoptosis in response to *T. gondii* infection.

## Background

As a typical intracellular protozoan parasite of warm-blooded vertebrates, *Toxoplasma gondii* has elaborate mechanisms to counteract host-cell apoptosis in order to maintain survival and breed in the host cells [1-4]. Upon invasion of *T. gondii*, the parasite uses a specialized set of secretory organelles to inject parasite-derived effector molecules into its host cell [5]. These effector molecules can specifically modulate host cell gene expression to improve the ability of the parasite to infect and proliferate, via inhibition of host immune responses, and change their gene expression to escape immunologic surveillance [6]. Quantitative analysis of the host mRNAs and proteome during *T. gondii* infection showed that upwards of 15% of mRNAs and upregulation of 213 protein spots display altered abundance relative to uninfected cells, including crucial mRNAs and proteins that were associated with the apoptotic pathway [7,8].

MicroRNAs (miRNAs) are endogenous small (19 ~ 24 nt long) noncoding RNAs that regulate gene expression in a sequence specific manner. This is primarily accomplished through binding to 3′UTR of target mRNAs, either targeting the transcripts for degradation or blocking their translation [9]. However, molecular mechanisms underlying miRNA gene transcriptional regulation are largely unclear. Recent studies on expression of miRNA genes have revealed potential transcriptional regulation by transcription factors, such as p53, NF-κB and MAPK [6,10,11]. Recently, the signal transducer and activator of the transcription (STAT) signaling pathway has emerged as a major target of exploitation by *Toxoplasma*. Infection of mouse bone marrow-derived macrophage induces rapid and sustained activation of signal transducers and activators of transcription 3(STAT3) [12,13]. Although a number of miRNAs have been shown to be important components of the STAT3 in various cell types [14-16], the potential interaction between miRNAs and STAT3 in human macrophage, in which the activation of STAT3 by infection with *Toxoplasma* may result in distinct biological consequences, has not yet been established.

In this study, we provide evidence that *Toxoplasma*-host cell interactions counteract the death of parasite-infected cells through upregulation of STAT3-binding miRNAs in human macrophage. The array analysis of miRNA exparession revealed significant alterations in miRNA expression in human macrophage following *Toxoplasma* infection. We report here that STAT3 mediated a prosurvival pathway by upregulation the miRNAs, leading to inhibition of host cells with *Toxoplasma* infection. Thus, the role of STAT3-binding miRNAs is postulated to be an important apparatus in *Toxoplasma* biology.

## Methods

### Parasites

The *T. gondii* strain, named TgCtwh3 with the atypical genotype China 1 (ToxoDB#9) and high virulence to mice as previously identified [17,18], was kept in the laboratory by mouse passage. Tachyzoites were maintained by twice weekly passage on HF (human fibroblast cells) in culture medium (DMEM with 10% FCS, 100U/ml penicillin and 100 μg/ml streptomycin, GIBCO).

### Human macrophage separation, culture and determination

Human PBMCs for *in vitro* parasite infection assays were isolated from the whole blood of 6 healthy individuals with written informed consent (2 female and 4 males, mean age 22+/-3 yrs). Ethical permission was obtained from the Institutional Review Board (IRB) of the Institute of Biomedicine at Anhui Medical University (Permit Number: AMU26-08610), which records and regulates all research activities in the school. Heparinized fresh whole blood (10 IU heparin/ml) was centrifuged against a Ficoll-Paque density gradient (density:1.077 g/ml; Solarbio) for 30 min at 2500 rpm. Peripheral blood mononuclear cells (PBMCs) were aspirated and washed in PBS before culture. The PBMCs were cultured at 37°C in 5% CO_2_ atmosphere at a density of 1 × 10^6^ cells/well in DMEM medium supplemented with 10% FCS. PBMCs were used as host macrophage after induction to differentiate with 1000U/ml recombinant human GM-CSF (Preprotech) for 48 h. Macrophages were stained with FITC-conjugated anti-CD14 to determine the purity of CD14^+^ cells. Control and infected cells were stained with Wright-Giemsa for 8 min, rinsed with distilled water and air dried. Cell morphology was observed by light microscopy. THP-1 cells (Cell bank, CAS) were cultured in DMEM medium supplemented with 10% FCS, and treated with 20 nmol of phorbol 12-myristate 13-acetate (PMA, Sigma) to induce THP-1 cells differentiation into macrophage-like THP-1 cells [19].

### MiRCURYTM LNA array analysis of miRNAs

The Exiqon (Vedbaek, Denmark) mercury LNA microRNA arrays service were used to process the samples were used. Briefly, human macrophage were exposed to TgCtwh3 for 24 h. Total RNAs from macrophage were harvested using TRIzol (Invitrogen) and miRNeasy min kit (QIAGEN) according to manual instructions. After having passed RNA quantity measurement using the NanoDrop 1000, the samples were labeled using the miRCURYTM Hy3TM/Hy5TM Power labeling kit and hybridized on the miRCURYTM LNA Array (v.16.0). Following the washing steps the slides were scanned using the Axon GenePix 4000B microarray scanner (Axon Instruments, Foster City, CA). Scanned images were then imported into GenePix Pro 6.0 software (Axon) for grid alignment and data extraction. Replicated miRNAs were averaged and miRNAs that intensities are ≥50 in all samples were chosen for calculating a normalization factor. Expressed data were normalized using the Median normalization. After normalization, differentially expressed miRNAs were identified through Volcano Plot filtering. Hierarchical clustering was performed using MEV software (v4.6, TIGR).

### qRT-PCR analysis

Macrophage were exposed to TgCtwh3 or LPS (1 μg/ml) as a control for different duration (6,12 and 24 h). For qPCR analysis of mature miRNAs, total RNA was reverse-transcribed from 0.05 μg total RNAs and determined with All-in-oneTM miRNA qRT-PCR detection kit (GeneCopoeia) by Applied Biosystems 7500 real-time PCR System (Applied Biosystems). The specific primers used in this reaction were as follows: miR-19b; miR-19a; miR-20a; miR-27b; miR-30c; miR-125 (all from GeneCopoeia).

For analysis of pri-miRNAs, total RNA was isolated from cells with Trizol reagent (Ambion). RNAs were treated with DNA-free TM Kit (Ambion) to remove any remaining DNA. Comparative real-time PCR was performed by using the SYBR Green PCR Master Mix (Applied Biosystems). Specific primers for pri-miRNAs were listed in Additional file 1: Table S1.

All reactions were run in triplicate. The Ct values were analyzed using the comparative Ct (ΔΔCt) method. Normalization was performed by using the small housekeeping U6 relative to the control (non-treated cells).

### Protein extraction and western blotting

To obtain total cellular lysates, transfected cells and controls were lysed in ice-cold cell lysis buffer [20 nM tris, 250 mM NaCl, 2 Mm EDTA, 0.1% Triton X-100, 0.01 mg/ml aprotinin, 0.003 mg/ml leupetin, 0.4 mM phenylmethylsulfonyl fluoride, and 4 mM NaVO_4_] (Sigma). The protein concentration was determined with the Bradford protein assay kit (BESTBIO) using a c-globulin standard curve. Proteins were separated by standard SDS-PAGE and transferred onto nitrocellulose membranes (Beyotime). Nonspecific binding sites were blocked using 5% dry skimmed milk, 0.2% Tween-20 in PBS (pH 7.4) for overnight at 4°C and then incubated with primary antibodies to STAT3 and GAPDH (Proteintech).

### Plasmid construction and transcriptional reporter assays

For construction of a constitutively active form of human STAT3, named STAT3-C, the amino acid residues at A661 and N663 were mutated to cysteine [20], the sequences were chemically synthesized and cloned into pEZ-M02 (GeneCopeia). STAT3-WT:3′-GAT**GCT**ACC**A AT**ATCCTGGT-5′; STAT3-C: 3′-GAT**TGC**ACC**TGC**ATCCTGGT-5′pEZ-M02-STAT3-WT (200 ng), pEZ-M02-STAT3-C (300 ng) or empty vector was individually cotransfected into THP-1 cells, together with appropriate miR-17 ~ 92 gene cluster promoter reporter plasmids (200 ng) by using Effectene Transfection Reagent (Qiagen). In each transfection, cells were also cotransfected with pRL-CMV (Renilla luciferase reporter plasmid, Promega). Firefly and Renilla luciferases activity was assayed using a Dual-Luciferase Reporter Assay System (Promega). The magnitude of activation values was measured relatively to the levels of Firefly luciferase activity in THP-1 cells transfected with empty vectors and normalized by Renilla luciferase activity. A mutation was introduced into the STAT3 binding site (STAT3-BR-1) using the following primers: sense 5′ - TAT GT**C CT**T GAG AAT TCC GGA ATT TCC TG - 3′; anti-sense 5′ - TCT CA**A GG**A CAT AAT TGT TAA AAG TGA GG - 3′ (alterations underlined) [20]. The correct sequence of each insert was confirmed by sequencing. SiRNA. THP-1 cells were transiently transfected with STAT3 siRNA or siRNA-control (40 nM, Santa Cruz) for 24 h using SuperFectin II *in vitro* siRNA Transfection Reagent (PuFei Biotech) according to the manufacturer’s protocols.

### Flow cytometry

Cell death following induction of apoptosis and infection with *Toxoplasma* was quantified by flow cytometry. Annexin-V/propidium iodide (PI) double assay was performed using the Annexin V-EGFP Apoptosis Detection Kit (BESTBIO). Infected or non-infected macrophages were washed twice with PBS. 1 × 10^6^ cells were resuspended in 400 μl binding buffer and stained with 5 μl Annexin V-EGFP according to the manual’s recommendations. 10 μl PI was added and allowed to incubate with cells for 5 min at 4°C in the dark. Then cells were analyzed using a FACSCalibur flow cytometer (BD Biosciences).

## Results

### *Toxoplasma* infection induces alterations in miRNA expression in macrophage *in vitro*

Human macrophage were separated from whole blood and cultured after 48 h (Additional file 2: Figure S1A). Cells were stained with FITC-conjugated CD14 antibody and subjected to flow cytometry to determine the purity of macrophage. The percentages of CD14^+^ cells were 94.7 ± 0.60% after induction with GM-CSF in PBMCs (Additional file 2: Figure S1B). To determine if the parasites could infect macrophage, cells of control and infected cells were examined using Wright-Giemsa (Additional file 2: Figure S1C). After 24 h of infection, the tachyzoites can be seen in the cytoplasm of macrophage (Additional file 2: Figure S1D).

**Figure 1:**
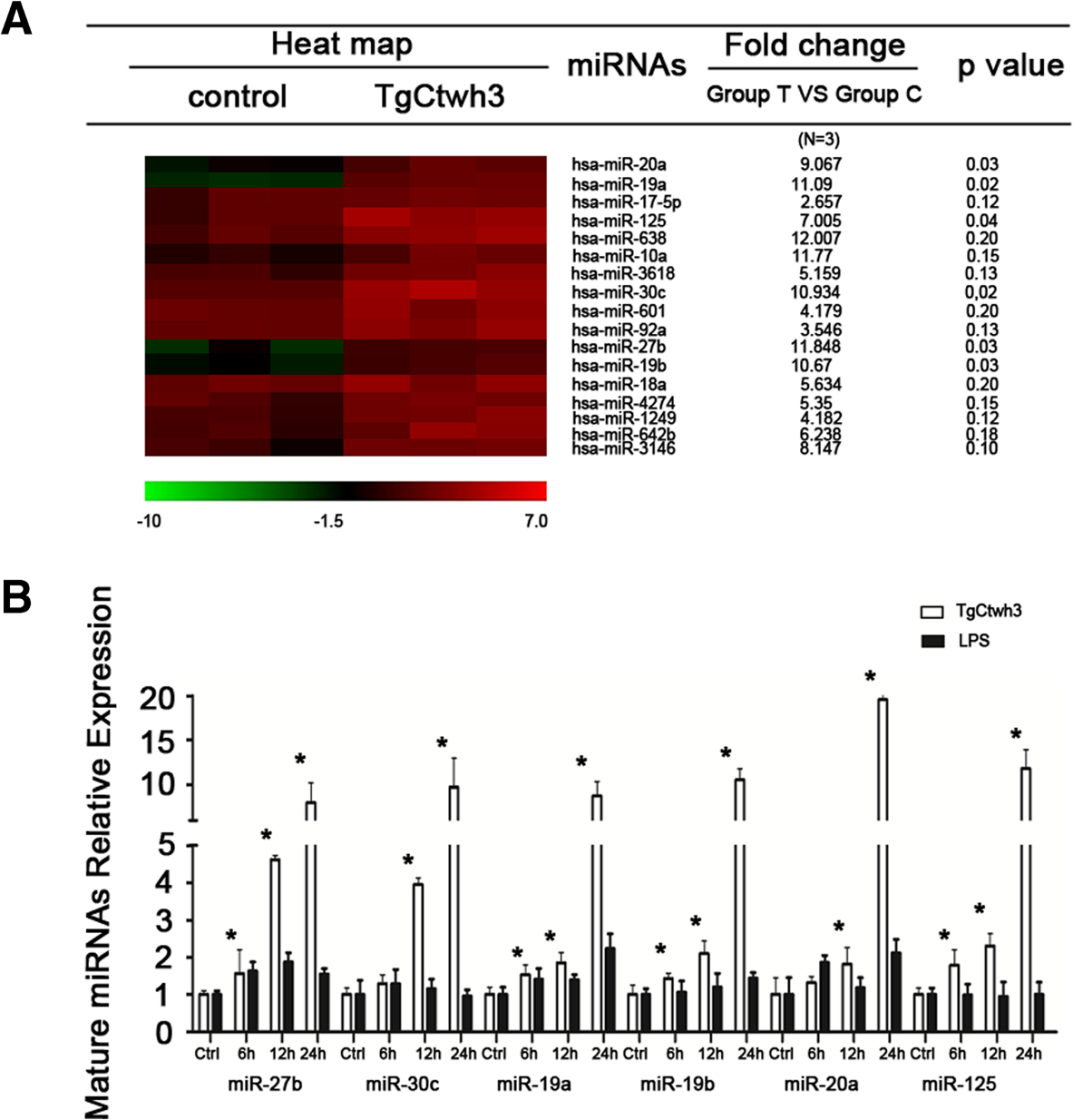
**Expression profiling of mature miRNAs in macrophage following *Toxoplasma *infection and LPS stimulation. (A)** miRNA expression profile in human macrophage following *Toxoplasma* infection. The left panel shows a heat-map of selected miRNAs indicating alteration in expression in macrophages following *Toxoplasma* infeciton.The filtered miRNA array data were subjected to unsupervised hierarchical clustering analysis. The metric was set as the Euclidean distance. The right panel shows expression miRNAs in macrophage following *Toxoplasma* infection. The fold change contains the ratio of mormalized intensities between *Toxoplasma*-infected macrophage (Group T) and non-infected macrophage (Group C). The p values are from the T test. **(B)** Altered expression of selected miRNAs confirmed by qRT-PCR. The amount of mature miRNAs was obtained by normalizing to the level of snRNA RNU6B in the samples. Graphics indicate those miRNAs showing an increased expression (including those significant change when *p*< = 0.05) in cells after treatment with LPS (n = 3) or exposure to *Toxoplasma* (n = 3). *, p,0.05 vs. the non-treated control.

To globally assess miRNA expression in human macrophage following *Toxoplasma* infection, we performed a microarray analysis of mature miRNA expression in macrophage. We profiled the levels of miRNAs extracted at 24 h from uninfected and tachyzoites-infected human macrophage using miRCURYTM LNA Array (v.16.0). A total of 17 miRNAs were upregulated (*p*< = 0.20) following *Toxoplasma* infection (Figure 1A). Of the miRNAs expressed, miR-20a, miR-125, miR-19a, miR-19b, miR-27b and miR-30c expression were significantly increased (*p*< = 0.05) in human macropahge after exposure to *Toxoplasma* infection for 24 h (Figure 1A).

To validate the microarray data and to specifically measure the effects of *Toxoplasm*a infection on miRNAs, qRT-PCR analysis using primers for mature miRNAs was performed to assess the kinetics of miRNAs in human macrophage following *Toxoplasma* infection. Increased expression of miR-20a, miR-125, miR-19a, miR-19b, miR-27b and miR-30c were noted in human macrophage at 6 h and 12 h postinfection, the abundance of these miRNAs significantly increased by ~23.5-fold at 24 h postinfection. The qRT-PCR analysis of miRNAs was also performed on human macrophage treated with LPS (1 mg/ml for 6 h, 12 h and 24 h) in order to determine the specificity of upregulation and expression of these miRNAs in *Toxoplasma*-infected cells. The results showed that increased expression of miRNAs was identified in *Toxoplasma*-infected cells but not in cells exposed to LPS at 24 h (*p* < =0.05; Figure 1B). No LPS contamination in the *Toxoplasma* preparation was detected using the Limulus Amebocyte Lysate (LAL) test kit (Bio-Whittaker) (data not shown).

### Database analysis of upregulated miRNAs in human macropahge following *Toxoplasma* infection reveals potential STAT3 binding sites In their promoter elements

Differential alterations in the mature miRNA expression profile of *Toxoplsma*-infected human macropahge suggest that miRNA gene expression is finely controlled in macropahge in response to *Toxoplasma* infection. One potential mechanism for selectively altering miRNA levels is through activation of distinct intracellular signaling pathways and nuclear transcription factors. Based on TFSEARCH (http://www.cbrc.jp/research/db/TFSEARCH.html) and MOTIF (http://motif.genome.jp/) database searches, many of these miRNA genes have putative STAT3 binding sites in their potential promoter elements (Table 1). We hypothesized that activation of the STAT3 pathway is involved in the transcription of selected miRNAs upregulated by *Toxoplasma*. Several miRNAs upregulated in human macropahge following *Toxoplasma* infection are cluster miRNAs; e.g., miR-19a, miR-19b and miR-20a are from the miR-17 ~ 92 gene cluster. So we focused on determining whether STAT3 binds to the promoter and transactivates the miRNA genes upregulated by *Toxoplasma* infection in human macropahge.

**Table 1:** **Analysis of *Toxoplasma*-upregulated miRNAs in human macrophage reveals potential transactivation of their genes by STAT3**

**Mature miRNAs**	**Chromosome (strand)**	**Host gene**	**Promoter**		Median (range) STAT3 binding score*
			**Start coordinates**	**End coordinates**	
miR-30c	1(+)	miR-30c-1	41173077	41177703	759(759–759)
		miR-30c-2			
miR-27b	9(+)	miR-23b-24-1	97763989	97769651	256(256–256)
miR-125	21(+)	miR-125b-1	17564208	17569306	348(348–348)
		miR-125b-2			
miR-19a	13(+)	miR-17 ~ 92	91995074	92000073	943 (943–943)
miR-19b					
miR-20a					

*MiRNAs with the highest STAT3 bindings scores according to the ENCODE ChIP-seq database are depicted.

### Differential expression of primary transcripts of *Toxoplasma*-upregulated mature miRNAs in human macrophage

**Figure 2:**
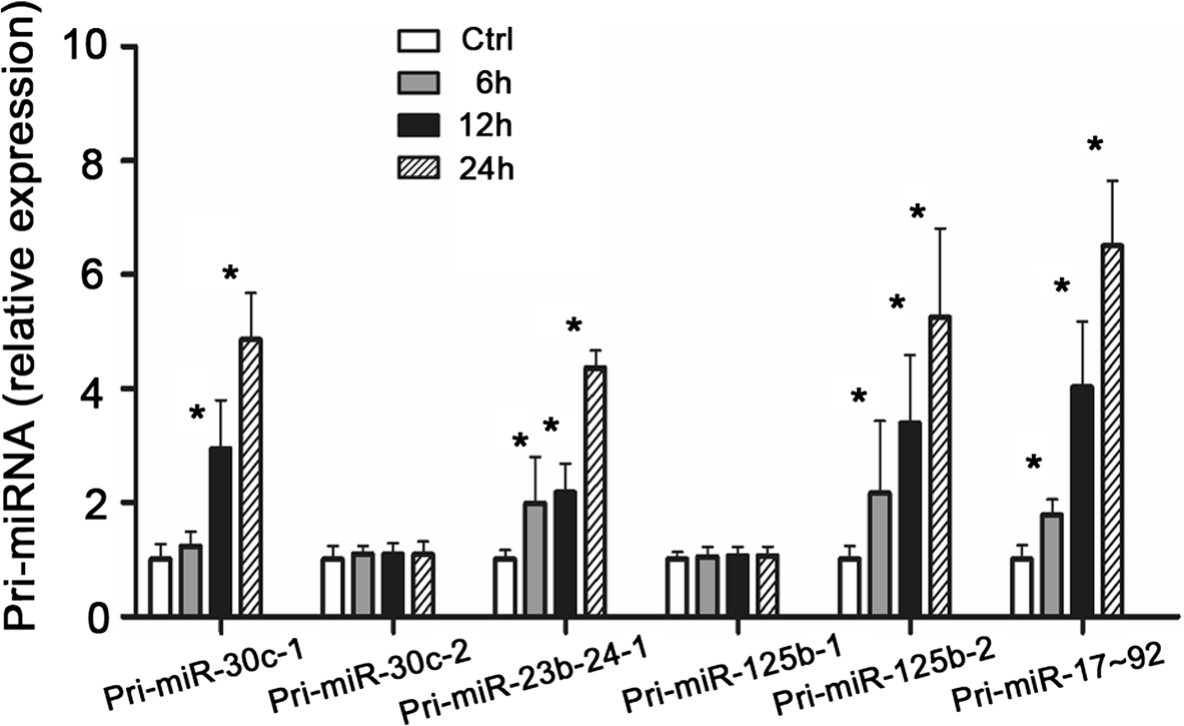
**Differential expression of primary transcripts of *Toxoplasma*-upregulated mature miRNAs in human macrophage.** Human macrophage were exposed to *Toxoplasma* for 6 h, 12 h and 24 h and primary transcripts (pri-miRNAs) of select miRNAs were quantified by qRT-PCR. The amount of pri-miRNAs was obtained by normalizing to the level of GAPDH in the samples. Data are expressed as the amount of pri-miRNAs in the infected samples relative to the control uninfected samples and representative of three independent experiments. *, p,0.05 vs. the non-infected control.

We then analyzed the kinetics of alterations of the primary transcripts (pri-miRNAs) for select mature miRNAs upregulated by *Toxoplasma* as listed in Table 1. Human macropahge were exposed to *Toxoplasma* for various periods of time and pri-miRNAs of interests were quantified by qRT-PCR (primers listed in Additional file 1: Table S1). Expression of pri-miR-30c-1, pri-miR-125b-2, pri-miR-23b-27b-24-1 and pri-miR-17 ~ 92 showed a time-dependent increase in cells following *Toxoplasma* infection (*p*< = 0.05, Figure 2). In contrast, for pri-miR-30c-2, pri-miR-125b-1, no significant increase was detected in cells after exposure to *Toxoplasma* infection (*p* > 0.05, Figure 2), suggesting a differential expression of the primary transcripts of *Toxoplasma*-upregulated miRNAs.

### Promoter binding of STAT3 is required for the transcription of select miRNA genes induced by *Toxoplasma* in human macrophage

**Figure 3:**
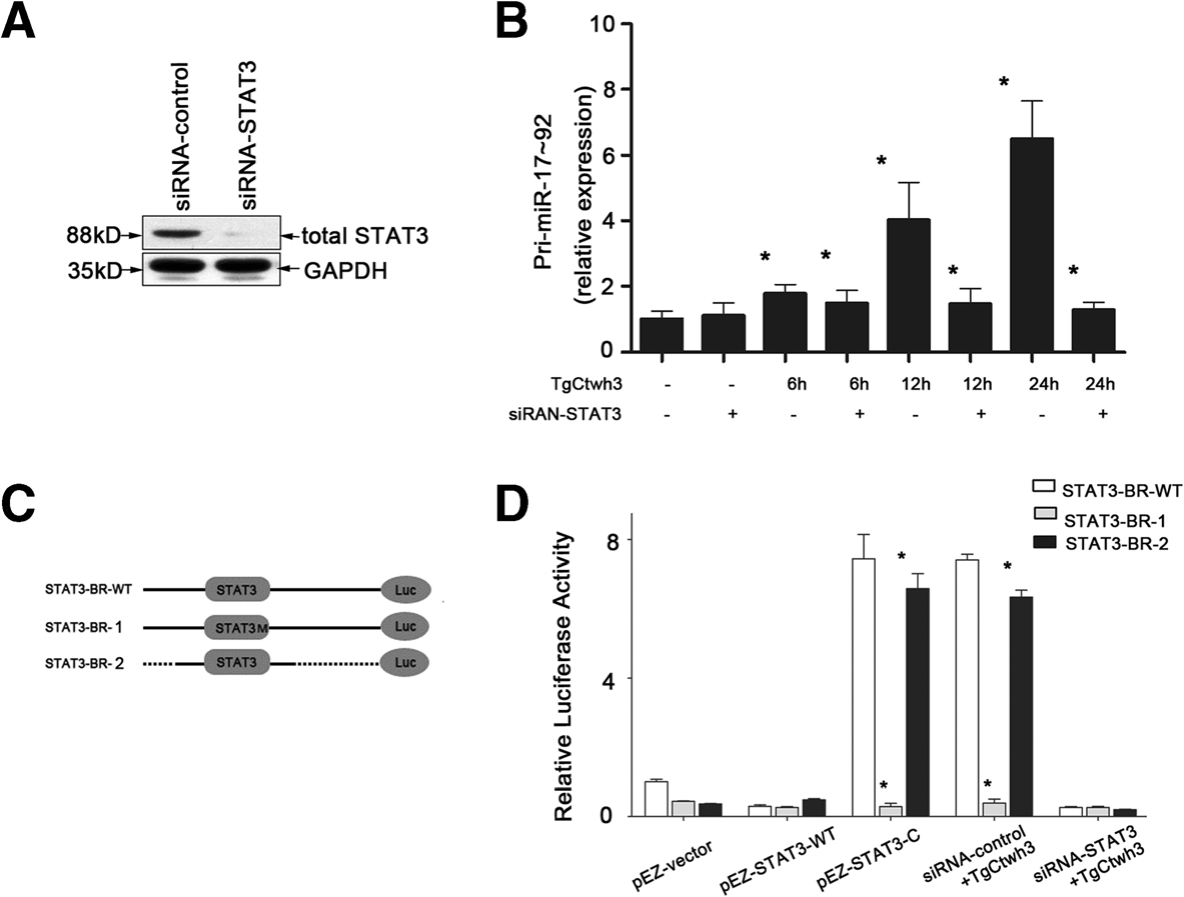
**Promoter binding of STAT3 transactivates miR-17 ~ 92 gene to increase mature miR-17 ~ 92 miRNAs expression following *Toxoplasma *infection. (A)** Western blot analysis showing stable knock-down of STAT3 by siRNA. **(B)** STAT3-dependent upregulation of pri-miR-17 ~ 92 in human macrophage following *Toxoplasma* infection. Data are presented as the relative expression level of pri-miR-17 ~ 92 in THP-1 cells following *Toxoplasma* infection in the presence or absence of siRNA as assessed by qRT-PCR. **(C)** A schematic illustration of pGL-based reporter constructs was used in dual luciferase assays to examine the transcriptional activity of the STAT3-BR in response to TgCtwh3 infection. Dotted lines indicated deleted region. **(D)** THP-1 cells were cotransfected with the indicated reporter constructs and Renilla luciferase plasmids. Twenty-four hours later, transcriptional activity was determined by luciferase assays. THP-1 cells with or without STAT3 knock-down by siRNA were cotransfected with the indicated reporter constructs and Renilla luciferase plasmids. Twenty-four hours later, cells were exposed to TgCtwh3 for another 24 h, followed by measurement of reporter activity by using luciferase assays. *, p,0.05 vs. non-treated cells (in **B**) or cells transfected with a control vector (in **D**) .Values are presented as mean ± SD (n =3 in **A**, **B** and **D**)

To test whether STAT3 is involved in *Toxoplasma*-induced transactivation of pri-miR-17 ~ 92 we exposed human macrophage to *Toxoplasma* infection in the presence of siRNA-STAT3, that prevents expression of the STAT3 (Figure 3A). siRNA-STAT3 blocked the *Toxoplasma*-induced increase of pri-miR-17 ~ 92 (Figure 3B). To further test the potential transactivation of miR-17 ~ 92 gene by STAT3, luciferase reporter gene constructs was used. *Toxoplasma* infection increased luciferase activity in cells transfected with the luciferase constructs that encompassed the binding site for STAT3 in the promoter region of miR-17 ~ 92 gene. A mutant of the STAT3 binding site blocked *Toxoplasma*-induced luciferase activity (Figure 3C). In addition, siRNA-STAT3 significantly inhibited the increase of luciferase activity induced by *Toxoplasma* infection (Figure 3D). Moreover, STAT3-associated transactivation of the miR-17 ~ 92 promoter was also confirmed by the upregulation of luciferase activity after STAT3 overexpression in the cells (Figure 3D). Together, these data demonstrate that STAT3 binding to the promoter element of the miR-17 ~ 92 gene mediates miRNAs (miR-17-5p, miR-18a, miR-19a, miR-20a, miR-19b and miR-92a) upregulation in human macrophage in response to *Toxoplasma* infection.

### Functional inhibition of selected STAT3-dependent miRNAs increase apoptosis of human macrophage with *Toxoplasma* infection

**Figure 4:**
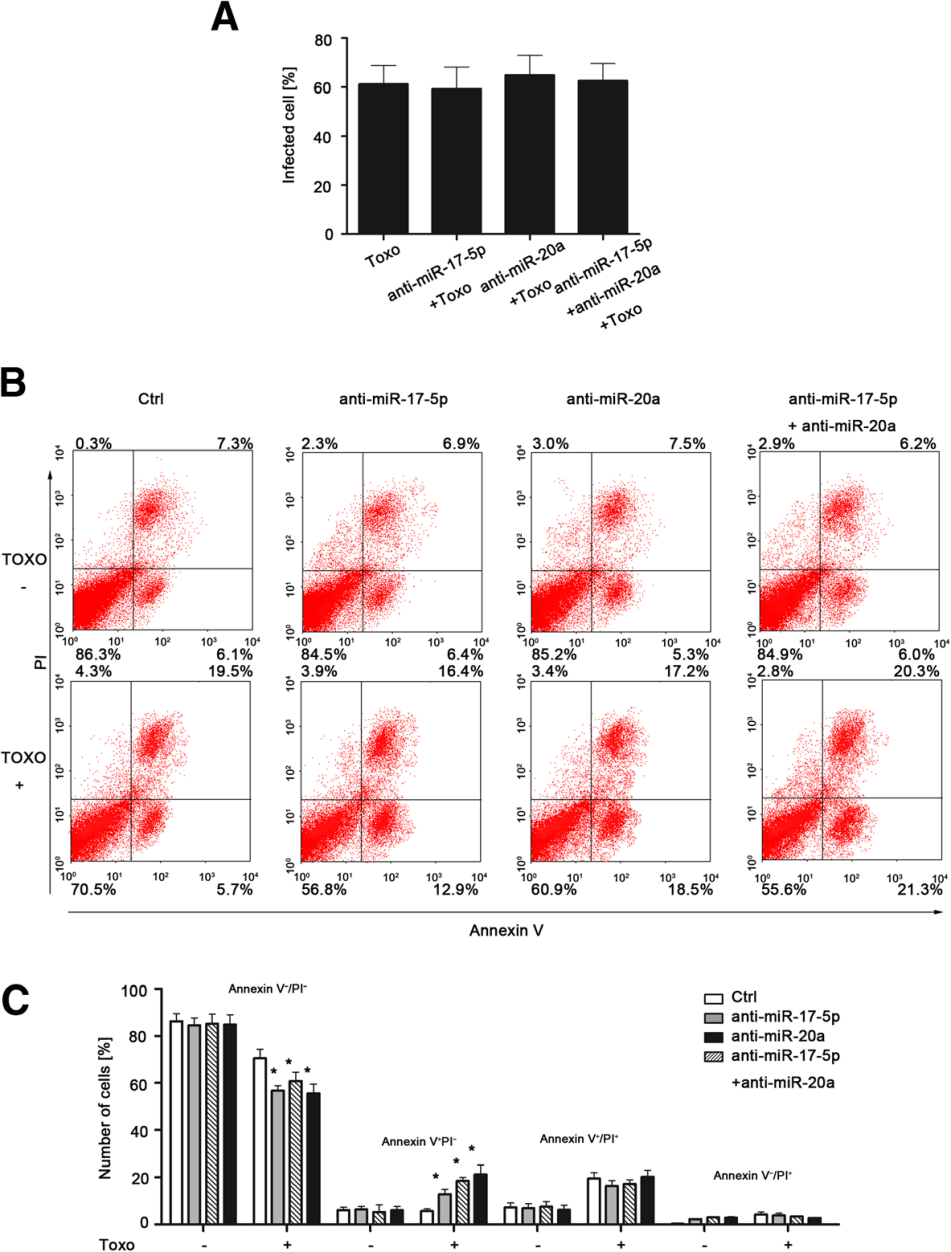
**Functional inhibition of selected STAT3-dependent miRNAs in human macrophage increases apoptosis of cells in vitro. (A)** Percentages of parasite-positive macrophage (Wright-Giemsa stain as shown in Additional file 2: Figure S1) after transfection with specific anti-miRs (black bars) or untreated (white bars). **(B)** Transfection of cells with anti-miRs on apoptosis of human macrophage for 24 h after initial exposure to the parasite. Dot plots depict the amounts of macrophage stained with Annexin V and/or PI; numbers indicate percentages of cells in each quadrant from a representative experiment. **(C)** Percentage of cells with *Toxoplasma* after transfected with anti-miR-17-5p, anti-miR-20a, or ant-miR-17-5p and anti-miR-20a. *, p,0.05 vs. the non-transfected with *Toxoplasma* infection control.Values are presented as mean ± SD (n = 3 in **A-B**).

To test whether miRNAs are involved in human macrophage inhibition of apoptosis with *Toxoplasma* infection, we assessed apoptosis over time in cultured macrophage transfected with various anti-miRs thereby inhibiting function of specific *Toxoplasma*-upregulated miRNAs. MiRNA inhibitors are commercially available, chemically modified single stranded nucleic acids designed to specifically bind to and inhibit endogenous miRNAs. Cells were transfected with specific anti-miRs (30 nM, Ambion) or a mixture of anti-miRs to miR-17-5b and miR-20a (a total of 30 nM with 15 nM for each), and then exposed to *Toxoplasma* for 24 h. The percentages of parasite-positive macrophage following exposure to *Toxoplasma* for 24 h was similar in all cultures, to those transfected with the specific anti-miRs (*p* > 0.05, Figure 4A), suggesting that those transfected with anti-miRs did not affect the number of parasite-infected macrophage. Cells transfected with anti-miRs to miR-17-5b and miR-20a, as well as a mixture of two anti-miRs, displayed a decreased the number of Annexin V-/PI- macrophage (viable cells) as compared to control cells. In addition, these anti-miRs increased the number of Annexin V+/PI- macrophage (early apoptosis) (*p* < = 0.05, Figure 4B).

## Discussion

There is evidence that miRNAs play a critical role in the regulation of apoptosis [21,22] A better understanding miRNA expression changes in human macrophage following *Toxoplasma* infection will provide new insights in miRNA-associated apoptosis. we report significant alterations in miRNA expression profiles in human macrophage following *Toxoplasma* infection. Our analysis of miRNAs upregulated by *Toxoplasma* in human macrophage revealed that miR-30c-1, miR-125b-2, miR-23b-27b-24-1 and miR-17 ~ 92 genes are transactivated via potential promoter binding of the STAT3.

These data provide several insights relevant to miRNA expression regulation in human macrophage following *Toxoplasma* infection. First, transactivation of miRNA genes that produce the same mature miRNA can be differentially controlled. Specifically, both mir-125b-1 and mir-125b-2 genes can produce mature miR-125b, but only transactivation of mir-125b-2 gene was detected in cells following *Toxoplasma* infection. In this study, we demonstrated that promoter binding of the STAT3 is required for transactivation of the miR-30c-1, miR-125b-2, miR-23b-27b-24-1 and miR-17 ~ 92 genes in cells following *Toxoplasma* infection. Given the complexity and variability in the gene structure for each miRNA, it is obvious that multiple mechanisms are involved in the transcriptional regulation of human miRNA genes [23-25]. Therefore, transcription of miRNA genes is expected to be a dynamic process in response to the constant alterations in intracellular signals. miRNA expression thus reflects the final integrated result of multiple interrelated signals on miRNA transcription. In this regard, other transcription factors, such as NF-kB, AP-1, c-myc, C/EBPa, may also be involved in the transcriptional regulation of miRNA genes in human macrophage in response to *Toxoplasma* infection. Future studies will focus on whether the other transcription factors are involved in the *Toxoplasma*-induced upregulation or downregulation of miRNA expression. Secondly, transactivation of genes of cluster miRNAs or as introns in other gene alleles may be controlled by the same promoter element. Of note, miR-19b, miR-19a and miR-20a are cluster gene miRNAs and co-transcribed with a host gene, C13orf25 [26]. *Toxoplamsa* infection upregulates expression of the mature forms of these three miRNAs, as well as pri-miR-17 ~ 92 and the host gene transcript, C13orf25. Our data are consistent with recent studies demonstrating transcriptional control of genes that code cluster miRNAs or that encode both miRNAs and other host transcripts [25]. Finally, the miR-17 ~ 92 gene appear to attenuate apoptotic responsiveness by targeting several mRNAs encoding pro-apoptotic effectors and favor progression from G1 to S-phase by targeting mRNAs that encode negative regulators of the cell cycle [27,28]. In our study, we found promotion of apoptosis of human macrophage with *Toxoplasma* infection via inhibition of miR-17 ~ 92 gene.

Previous investigation showed that a single amino acid substitution in the kinase domain of *Toxoplasma* ROP16 in difference strains determined STAT3 activation between type I and type II. Significantly, we found that TgCtwh3 strain with atypical genotype China 1 (ToxoDB#9) has the same amino acid substitution of the kinase domain of ROP16 at 503 bp (503 L, data not shown) as that of TGGT1 strain (503 L) with archetypical type I, which might account for the identical way of activation to STAT3 as type I [12]. Although *Toxoplasma* can infect any kind of nucleated cells, macrophage and related mononuclear phagocytes are its preferred target *in vivo*, and the parasite seems to have multiple ways of avoiding being killed [29,30]. Therefore, macrophage-centric research is essential to understand the basic strategies of the parasite-host interaction [31]. miRNAs have been identified in both mammalian and nonmammalian cells including virus and parasites [32,33]. *Toxoplasma* could increased the levels of miRNA in HFF cells [34], however, expression of miRNAs in *Toxoplasma* has not yet been examined and whether *Toxoplsma*-derived miRNAs can be localized in human macrophage is unknown. Nevertheless, the probes used in the microarray analysis in this study are human-miRNA specific with minimal cross-interaction with known miRNAs from other species.

## Conclusions

In summary, this first miRNA profiling in human macrophage in response to *Toxoplasma* infection in vitro revealed significant alterations in cellular miRNA expression. The mechanism by which *Toxoplasma* induces upregulation of a panel of miRNAs in human macrophage involves transactivation of miRNA genes through promoter binding of the STAT3. In addition, functional inhibition of the upregulated miRNAs increases apopotosis of macrophage with *Toxoplasma* infection thereby implicating these miRNAs in host cell help parasite survival. These data demonstrate a better understanding of miRNA expression in human macrophage following *Toxoplasma* infection will play a critical role in inhibition of apoptosis of host cells and it may provide new insights into relationship between the intracellular parasite and its hose cells.

## Competing interests

The authors declare that they have no competing interests.

## Authors’ contributions

JLS, YHC and LJ conceived and designed the study. YHC, HC and YBY performed the experiments. YHC and HC analyzed the data. YHC and JLS drafted the manuscript. All authors read and approved the final manuscript.

## Supplementary Material

Additional file 1: Table S1 Primers used for qRT-PCR. Click here for file

Additional file 2: Figure S1 Determination of macrophage from PBMCs. PBMCs-derived macrophage were separated from whole blood and stained with FITC-conjugated CD14 antibody to determine the purity of macrophage. (A) PBMCs-derived macrophage under microscope (×200). (B) CD14 expression of PBMCs-derived macrophage. (C) PBMCs-derived macrophage seen by microscopy (Wright-Giemsa, ×1000). (D) PBMCs-derived macrophage with TgCtwh3 for 24 h seen by microscopy (Wright-Giemsa, ×1000). Click here for file
