# Supplementary material for: STAT3-dependent transactivation of miRNA genes following Toxoplasma gondii infection in macrophage
Source: Parasit Vectors. 2013 Dec 16;6:356. doi: 10.1186/1756-3305-6-356 (PMC3878672; doi:10.1186/1756-3305-6-356)
Supplement: Additional file 1: Table S1 — Primers used for qRT-PCR. [file 1756-3305-6-356-S1.doc]

**Table S1 Primers used for qRT-PCR**

| Pri-miRNA | Sense primer(5′-3′) | Antisense primer(5′-3′) |
| --- | --- | --- |
| Pri-miR-30c-1 | CTGTGGGCTATAACCATGCTGTAG | GATCTGCGGAGTGGAGACTGTT |
| Pri-miR-30c-2 | CCTAGAGAGCACTGAGCGACAGA | TTCTCCCAGCTTTCTTACTTTCCA |
| Pri-miR-23b-24-1 | TCACATTGCCAGGGATTACCA | TGCACCTGTTCTCCAATCTGC |
| Pri-miR-125b-1 | CCATACCACCTGTTTGTTGCATCT | CTGAGAGGAGCGCAACAATGT |
| Pri-miR-125b-2 | GAAGAATTCTACCGCATCAAACCA | CTGCAGACAATCAATAAGGTCCAA |
| Pri-miR-17~92 | TTGCTAAGTGGAAGCCAGAAG | CATCCACGTGGCAAAACAT |
