# Supplementary figures and images for: STAT3-dependent transactivation of miRNA genes following Toxoplasma gondii infection in macrophage
Source: Parasit Vectors. 2013 Dec 16;6:356. doi: 10.1186/1756-3305-6-356 (PMC3878672; doi:10.1186/1756-3305-6-356)

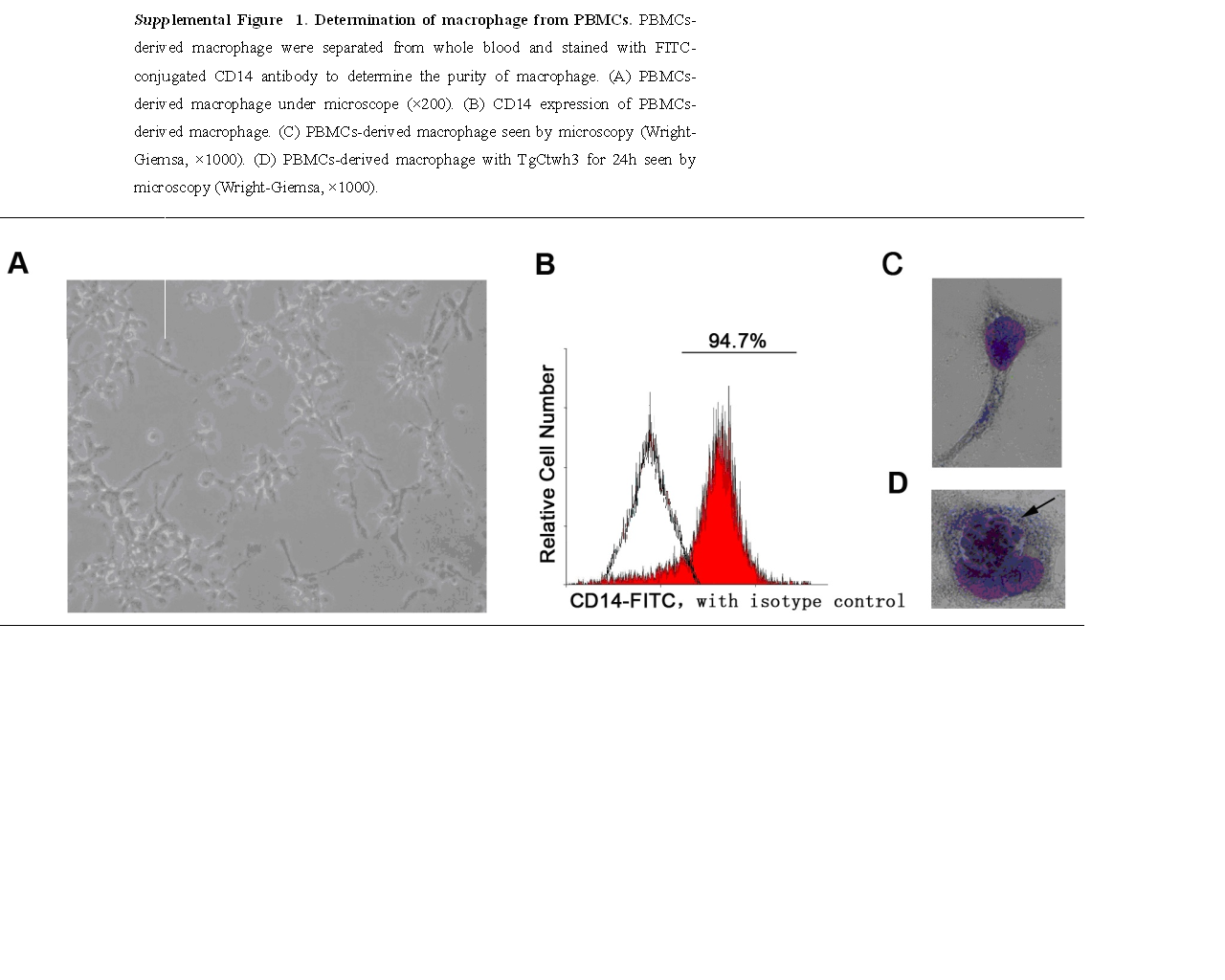

Supplement: Additional file 2: Figure S1 — Determination of macrophage from PBMCs. PBMCs-derived macrophage were separated from whole blood and stained with FITC-conjugated CD14 antibody to determine the purity of macrophage. (A) PBMCs-derived macrophage under microscope (×200). (B) CD14 expression of PBMCs-derived macrophage. (C) PBMCs-derived macrophage seen by microscopy (Wright-Giemsa, ×1000). (D) PBMCs-derived macrophage with TgCtwh3 for 24 h seen by microscopy (Wright-Giemsa, ×1000). [file 1756-3305-6-356-S2.bmp]
